# Supplementary material for: Tankyrase inhibition preserves osteoarthritic cartilage by coordinating cartilage matrix anabolism via effects on SOX9 PARylation
Source: Nat Commun. 2019 Oct 25;10:4898. doi: 10.1038/s41467-019-12910-2 (PMC6814715; doi:10.1038/s41467-019-12910-2)
Supplement: Supplementary file 3 — Supplementary Information [file 41467_2019_12910_MOESM3_ESM.docx]

SUPPLEMENTARY INFORMATION

**Tankyrase inhibition preserves osteoarthritic cartilage by coordinating cartilage matrix anabolism via effects on SOX9 PARylation**

Kim et al.

**Supplementary Figures 1-9**

**Supplementary Tables 1-11**

**Supplementary figures and figure legends**

**
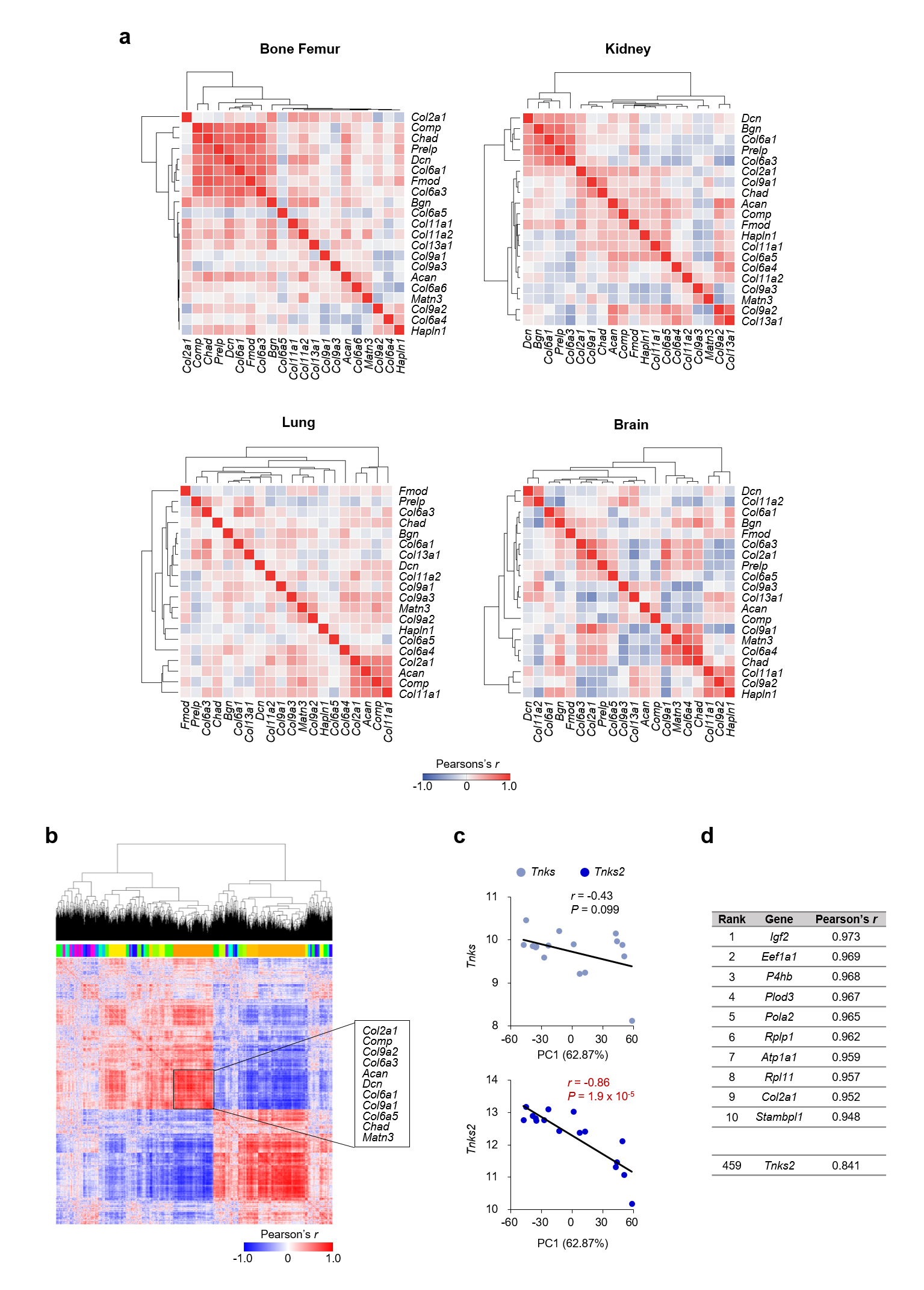
**

**Supplementary Fig. 1** Gene co-expression analysis using BXD cartilage transcriptome.

**a** Cartilage matrix genes are not inter-correlated in non-cartilaginous organs. Heatmaps of Pearson’s correlation coefficients of transcript levels for cartilage matrix genes in bone femur, kidney, lung, and brain of the 16 BXD mouse strains. **b** Correlation heatmap of transcript levels of the 16,074 genes of the cartilage dataset. A black box in the heatmap indicates a highly correlated module enriched with cartilage matrix genes. **c** Correlation between *Tnks* or *Tnks2* mRNA levels and the first principal component (PC1) generated by principal component analysis conducted with the genes in the module. **d** Pearson’s correlation coefficients between Factor 1 and various genes with a potential regulatory function.

**
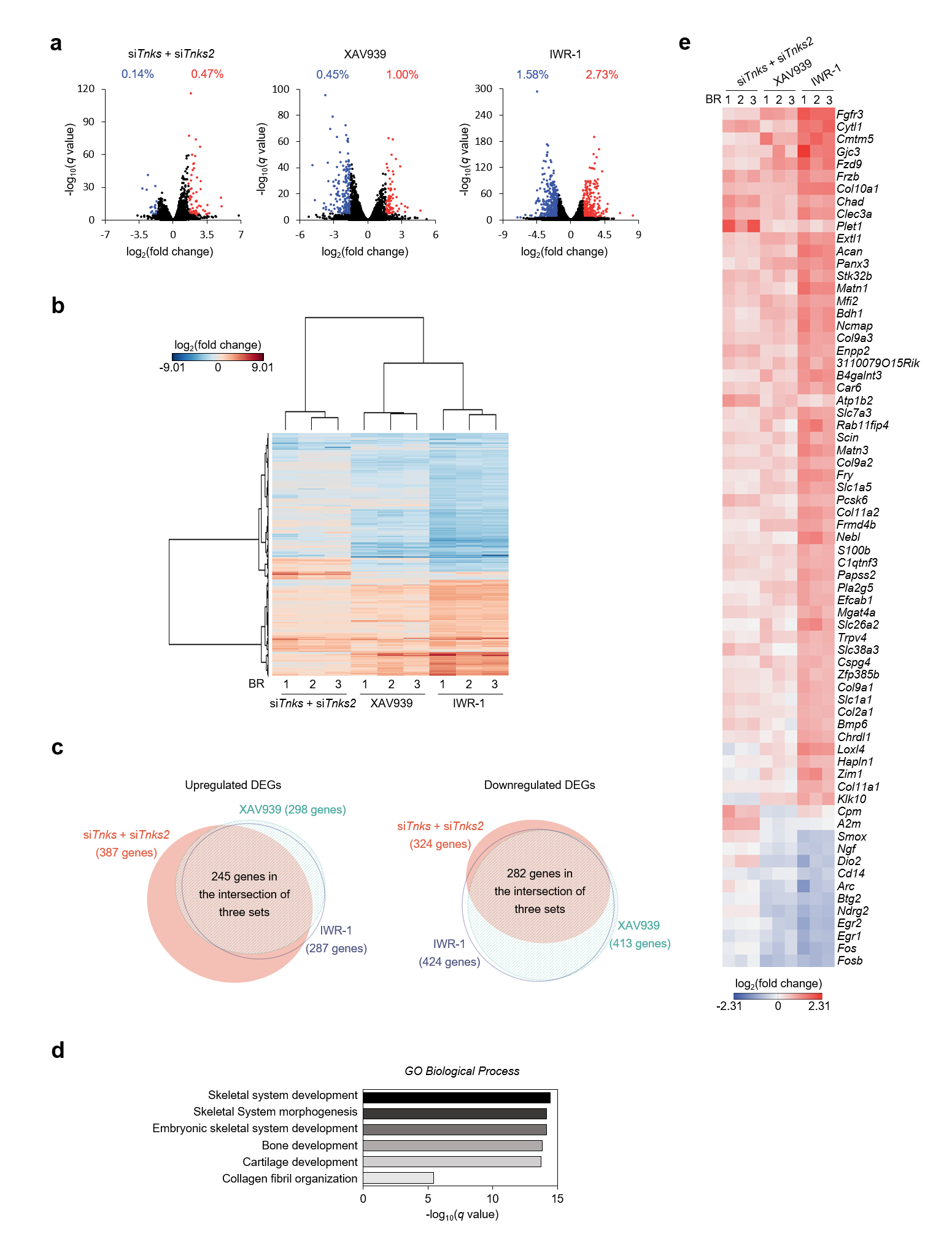
**

**Supplementary Fig. 2** Tankyrase inhibition elicits cartilage-specific transcriptomic profile.

**a** Volcano plots of gene expression changes in mouse chondrocytes treated with si*Tnks* and si*Tnks2* or tankyrase inhibitors. Red dots represent genes with a fold change of > 3 and a FDR *q* of < 1 × 10^-5^. Blue dots represent genes with a fold change of < $\frac{1}{3}$ and a FDR *q* of < 1 × 10^-5^. **b** Hierarchical clustering of fold changes of genes differentially expressed in chondrocytes in at least one condition (si*Tnks* + si*Tnks2*, XAV939, or IWR-1) compared to respective controls. RNA-Seq was conducted with three biological replicates (BR). **c** Venn diagrams for upregulated or downregulated DEGs of the three different tankyrase inhibition groups (si*Tnks* + si*Tnks2* vs. siControl, XAV939 vs. DMSO, and IWR-1 vs. DMSO). **d** GO analysis on differentially expressed genes upregulated in all three conditions (si*Tnks* + si*Tnks2*, XAV939, and IWR-1) in chondrocytes. **e** Fold change heatmap of cartilage-signature genes in mouse chondrocytes treated with si*Tnks* and si*Tnks2* or tankyrase inhibitors. List of cartilage-signature genes is provided in Supplementary Table 9.

**
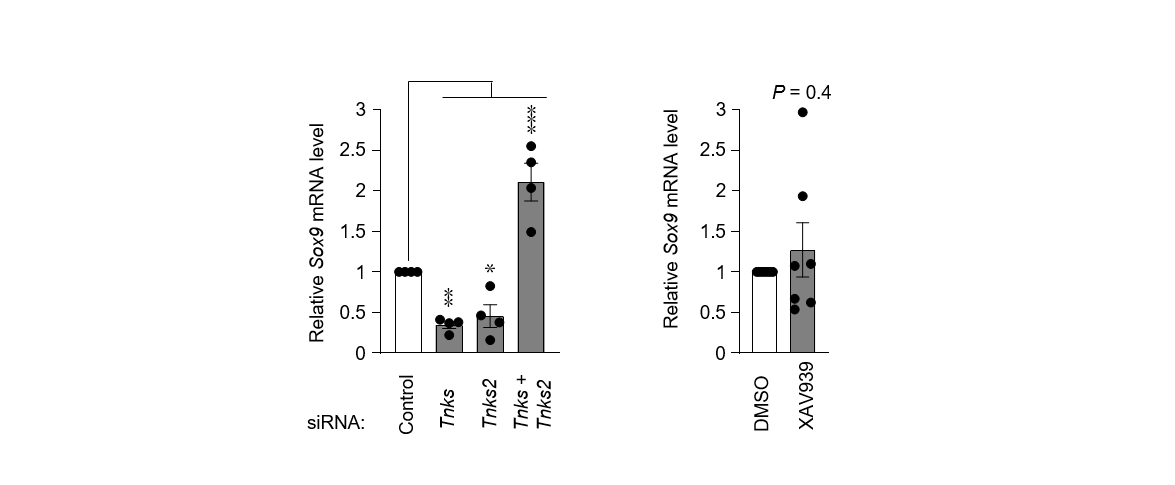
**

**Supplementary Fig. 3** The effect of tankyrase inhibition on *Sox9* transcript in chondrocytes

mRNA levels of *Sox9* in mouse chondrocytes treated with tankyrase siRNAs (*n* = 4) or XAV939 (10 μM, 108 h; *n* = 7). Data represent means ± s.e.m. **P* < 0.05, ***P* < 0.01, ****P* < 0.001; by ANOVA (left) or *t* test (right).

**
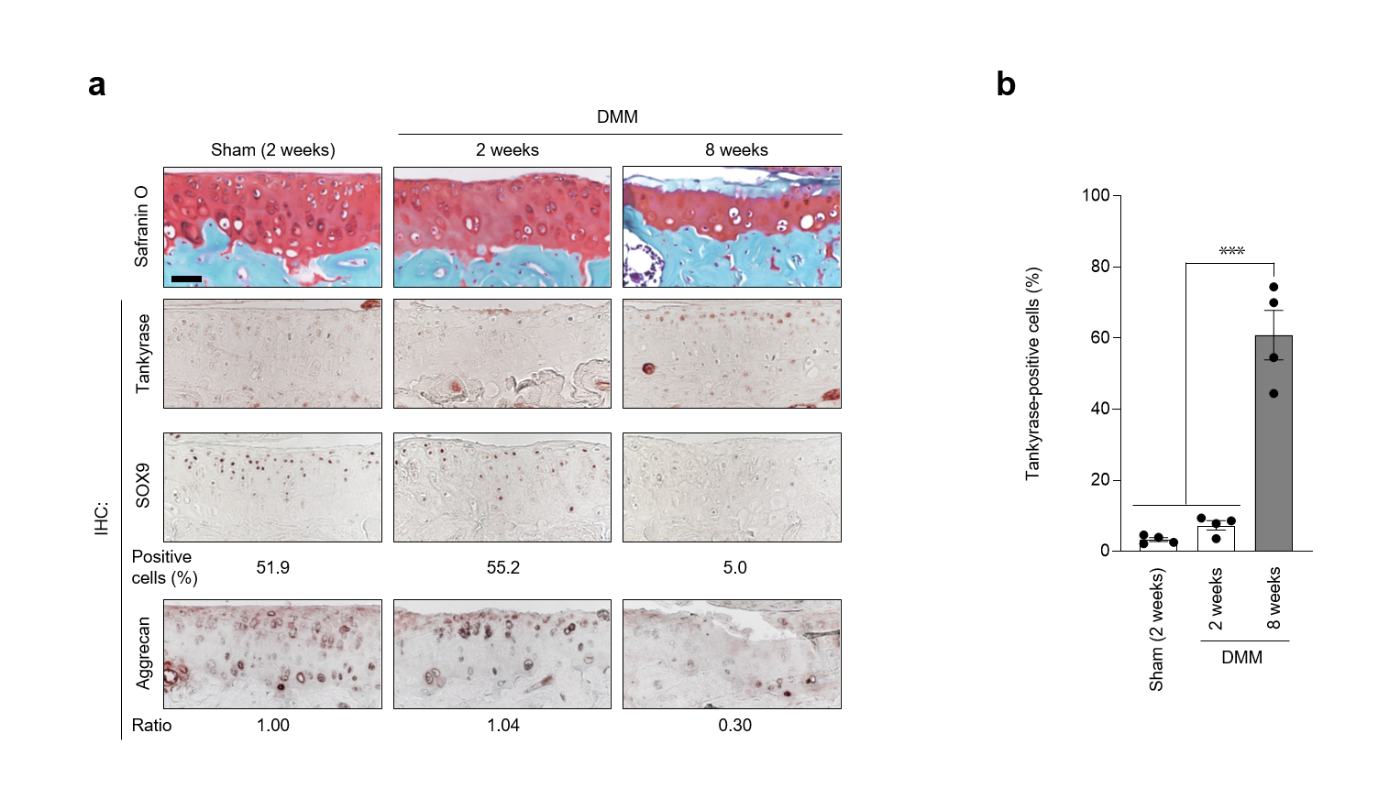
**

**Supplementary Fig.** **4** Tankyrase expression during OA development in mice.

**a** Safranin O staining and immunostaining for tankyrase and cartilage matrix proteins in the articular cartilage of DMM-operated mice. Scale bar: 50 μm. The relative chromogen intensity or the percentage of immunopositive cells is indicated. **b** Quantification of tankyrase expression by immunohistochemistry (*n* = 4). Data represent means ± s.e.m. ****P* < 0.001; by ANOVA.

**
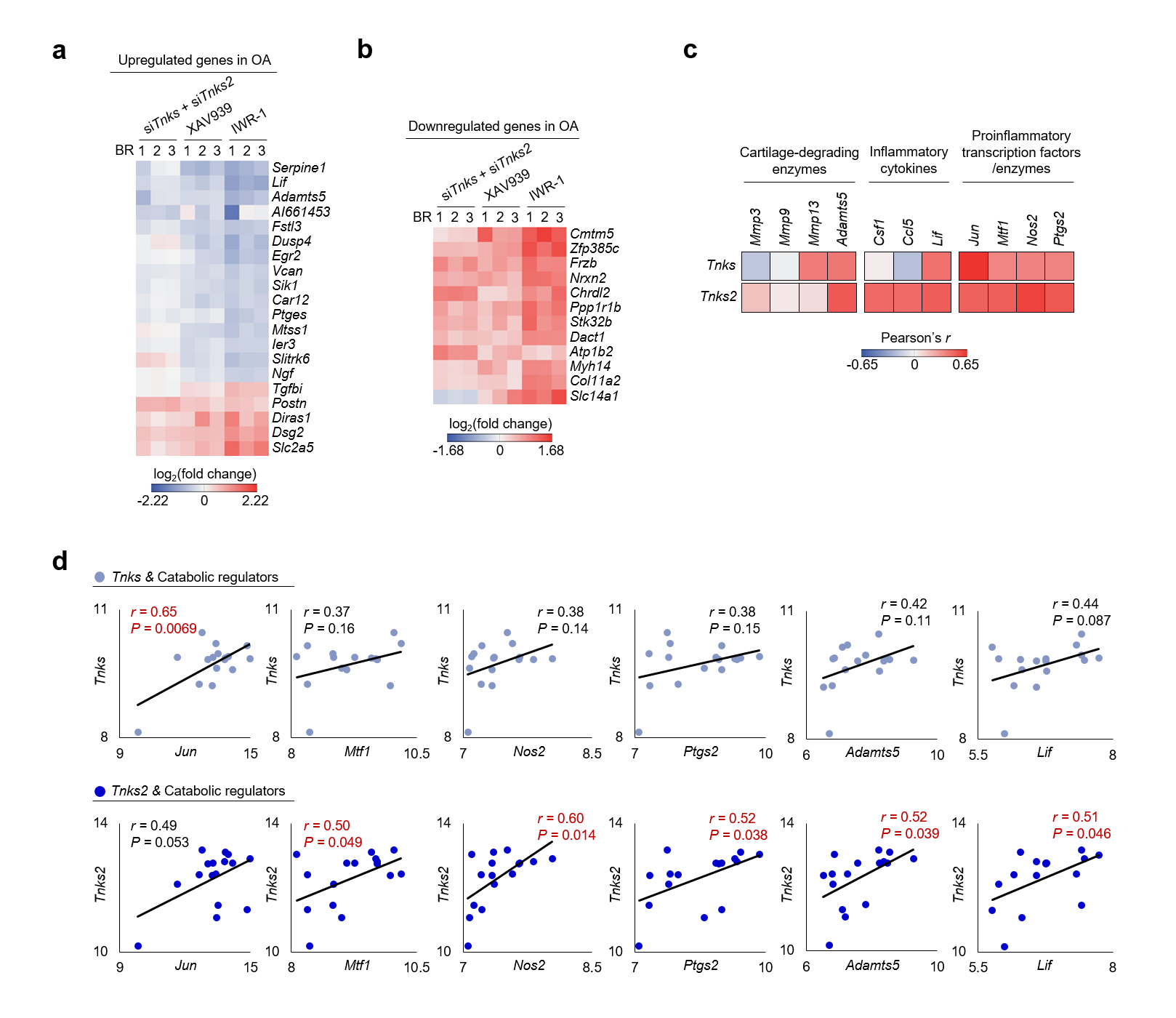
**

**Supplementary Fig. 5** Tankyrase inhibition inverts gene expression profiles associated with OA cartilage.

**a**, **b** Fold change heatmaps of OA-associated genes in mouse chondrocytes treated with si*Tnks* and si*Tnks2* or tankyrase inhibitors. Genes that are upregulated and downregulated in OA cartilage are listed in Supplementary Tables 10 and 11, respectively. **c** Heatmap of Pearson’s correlation coefficients between transcript levels of *Tnks* or *Tnks2* and catabolic genes in the articular cartilage of 16 BXD mouse strains. **d** Correlation between *Tnks* or *Tnks*2 and catabolic regulators mRNA levels in the articular cartilage of 16 BXD mouse strains.

**
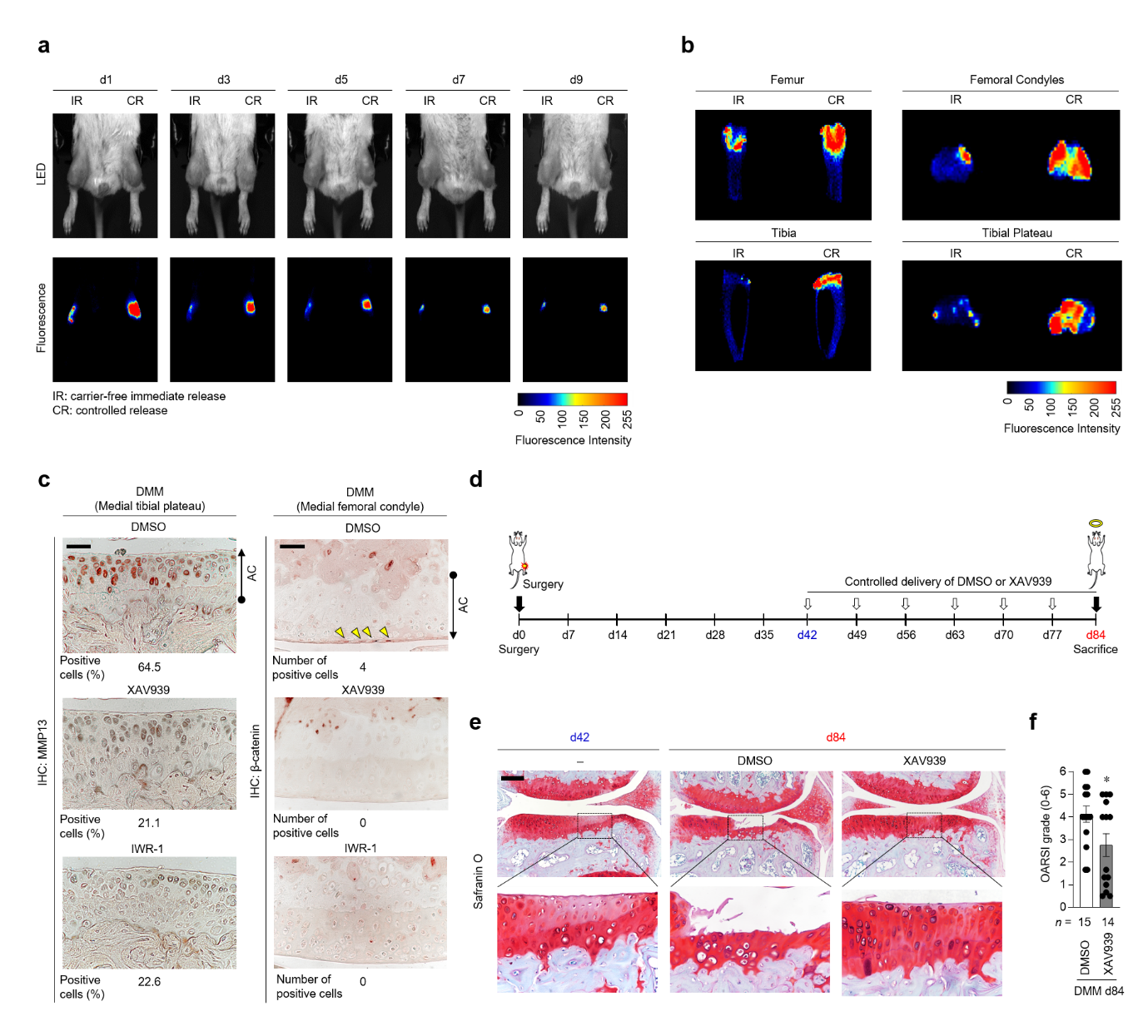
**

**Supplementary Fig. 6** Tankyrase inhibition prevents progression of OA.

**a** Light-emitting diode (LED) and fluorescence images of mouse knee joints intra-articularly injected with carrier-free DiD or DiD-loaded ascorbyl palmitate hydrogel. Images were acquired on the indicated days after injection. **b** Fluorescence images of mouse femur (femoral condyle) and tibia (tibial plateau) with carrier-free DiD or DiD-loaded ascorbyl palmitate hydrogel. Images were acquired at 9 days after IA injection. **c** Immunostaining of MMP13 and β-catenin in articular cartilage of DMM-operated mice. Scale bars: 50 μm. The percentage and the number of immunopositive cells are indicated. Arrowheads indicate chondrocytes that specifically express β-catenin in the superficial zone of articular cartilage. **d** Schematic representation of controlled drug delivery to DMM-operated mice. **e**, **f** Cartilage destruction assessed by (**e**) Safranin O staining (scale bar: 200 μm) and (**f**) OARSI grade. Data represent means ± s.e.m. **P* < 0.05; by Mann-Whitney test.

**
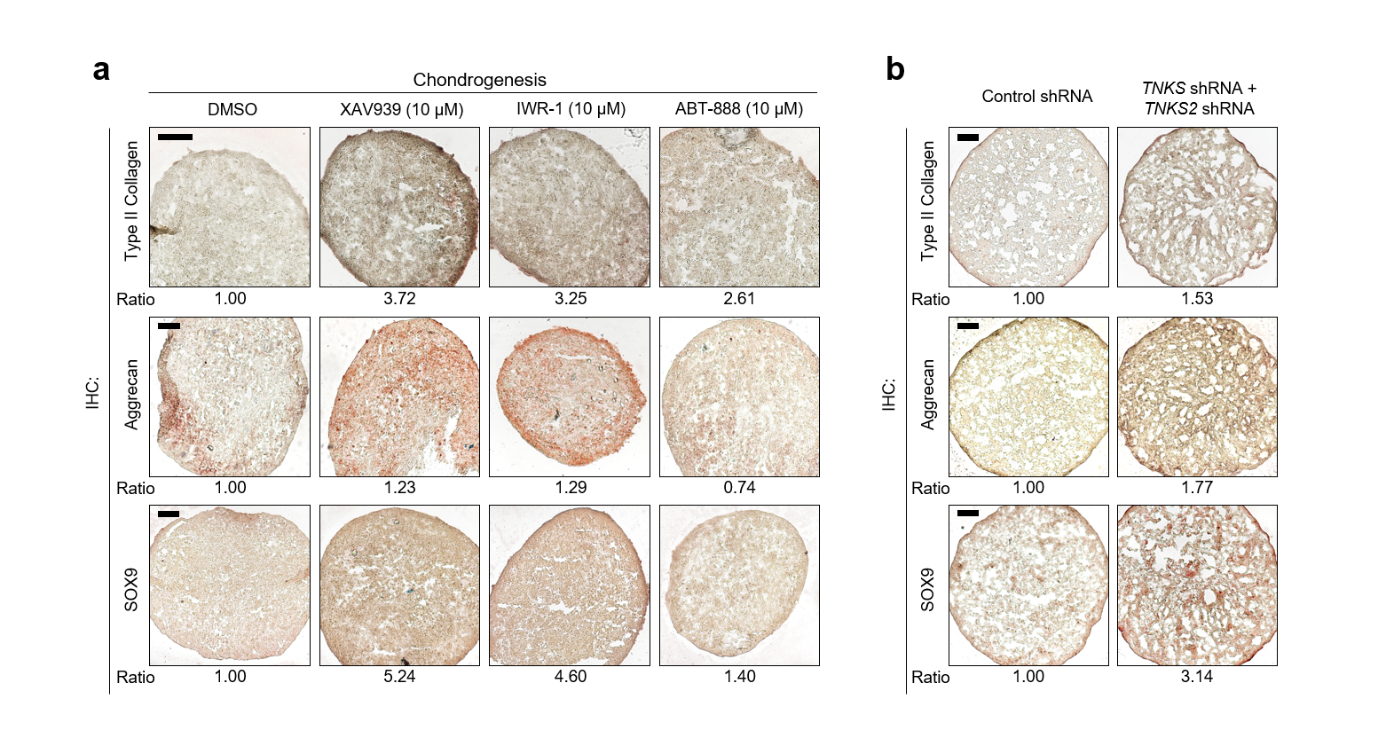
**

**Supplementary Fig. 7** Tankyrase inhibition enhances expression of chondrogenesis marker genes during chondrogenic differentiation of mesenchymal stem cells.

**a, b** Immunostaining of chondrogenesis marker genes in pellet cultured hMSCs treated with (**a**) the indicated drugs or (**b**) shRNAs. Scale bars: 100 μm. The relative chromogen intensity is indicated.

**
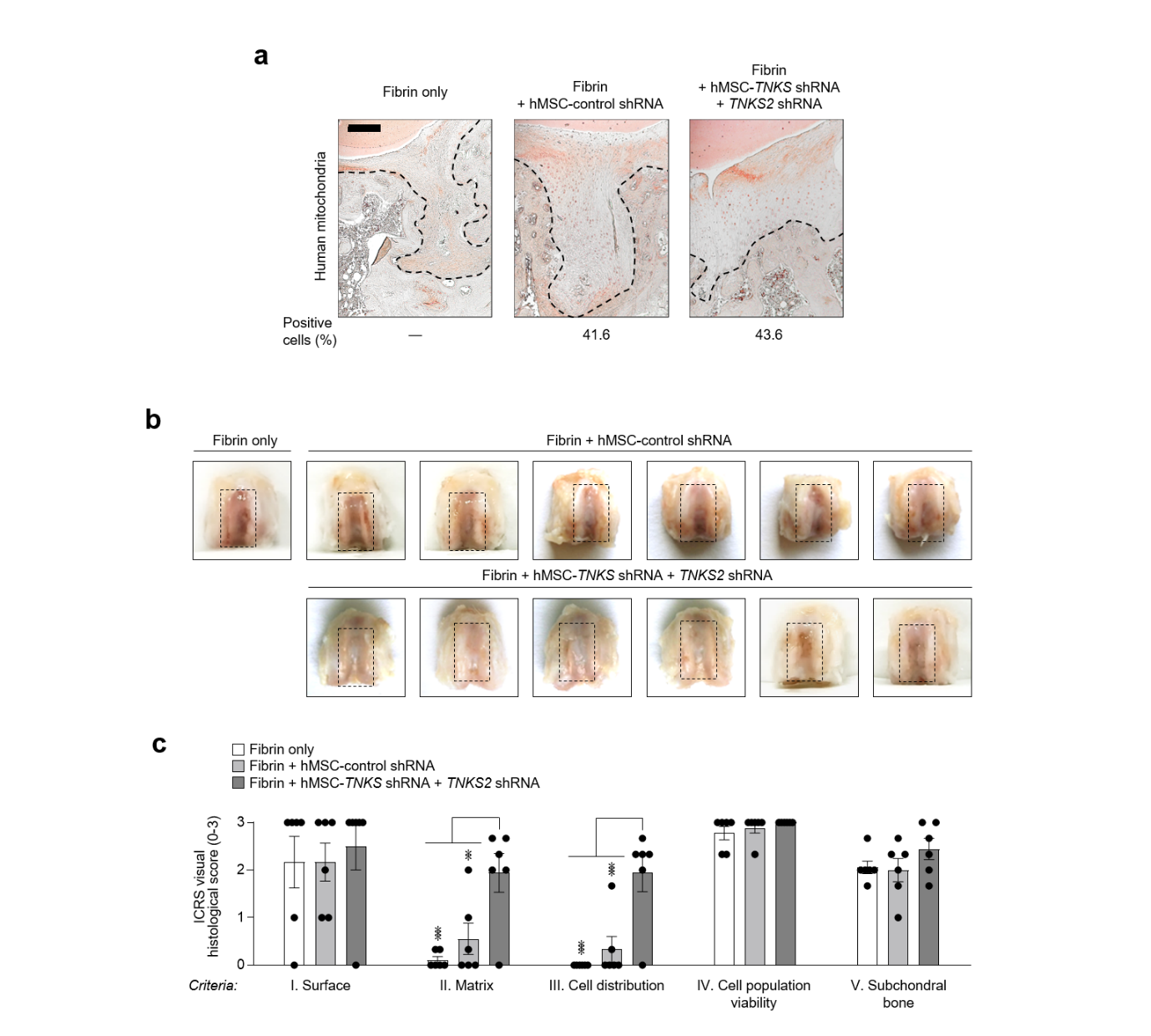
**

**Supplementary Fig. 8** Tankyrase inhibition stimulates chondrogenic differentiation of mesenchymal stem cells in vivo.

**a-c** hMSCs infected with control shRNA lentivirus or *TNKS* shRNA and *TNKS2* shRNA lentiviruses were implanted in the full-thickness cartilage lesions of rat knee joints with fibrin gel constructs. A fibrin-only group was used as a control. (**a**) Human mitochondrial immunostaining in cartilage lesions. The area above the dashed line indicates defect regions that have been implanted with fibrin gel only or fibrin gel containing hMSCs. Scale bar: 500 μm. The percentage of immunopositive cells is indicated. (**b**) Gross appearance of the indicated groups 8 weeks after transplantation. Transplantation of hMSCs with *TNKS* and *TNKS2* knockdown resulted in superior healing, filling lesions with cartilage-like tissues. (**c**) Cartilage repair was assessed using various criteria of the ICRS visual histological score system for in vivo repaired cartilage (*n* = 6). Data represent means ± s.e.m. ***P* < 0.01, ****P* < 0.001; by ANOVA.

**
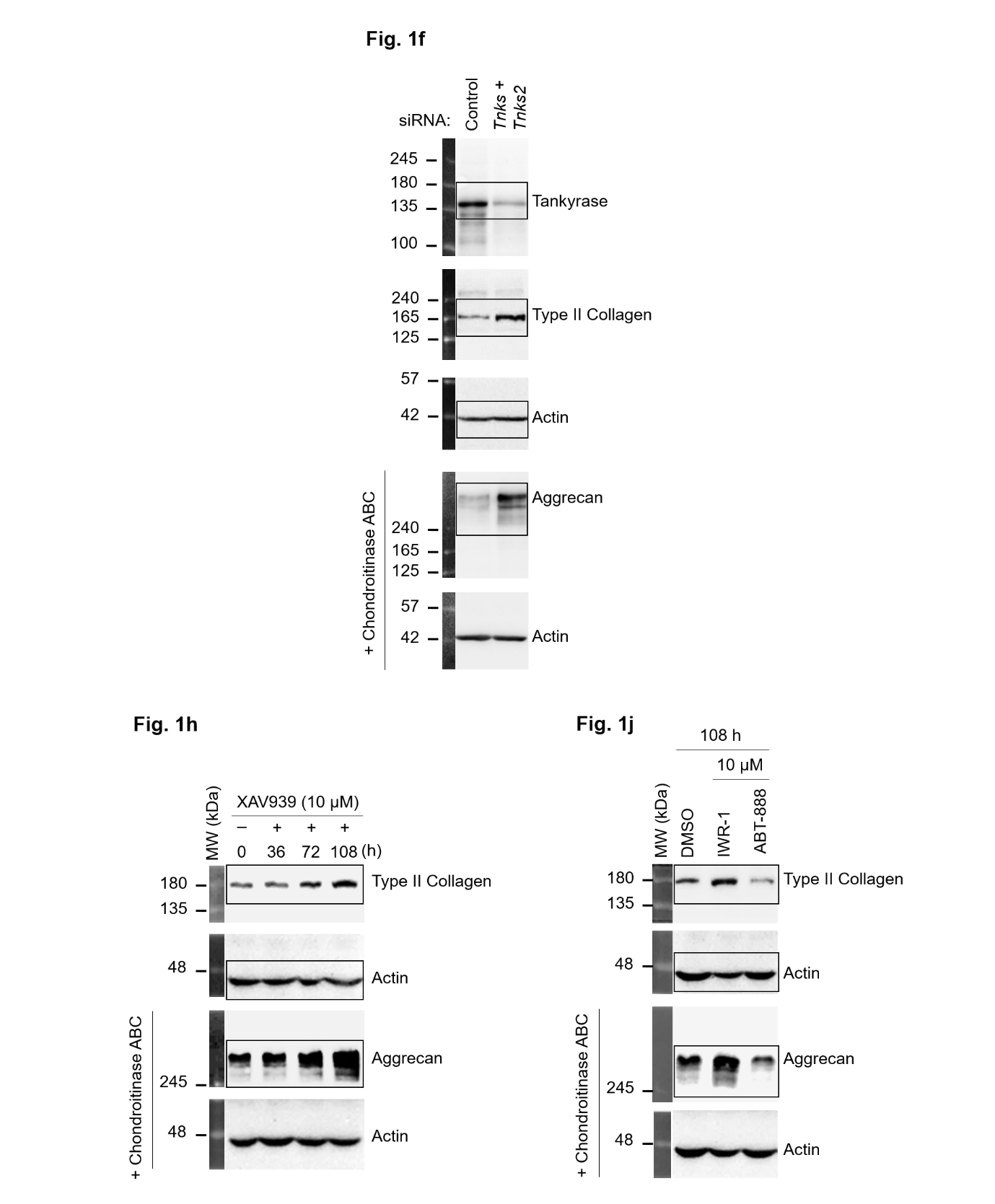
**

**Supplementary Fig. 9** Full-size immunoblot images.

**
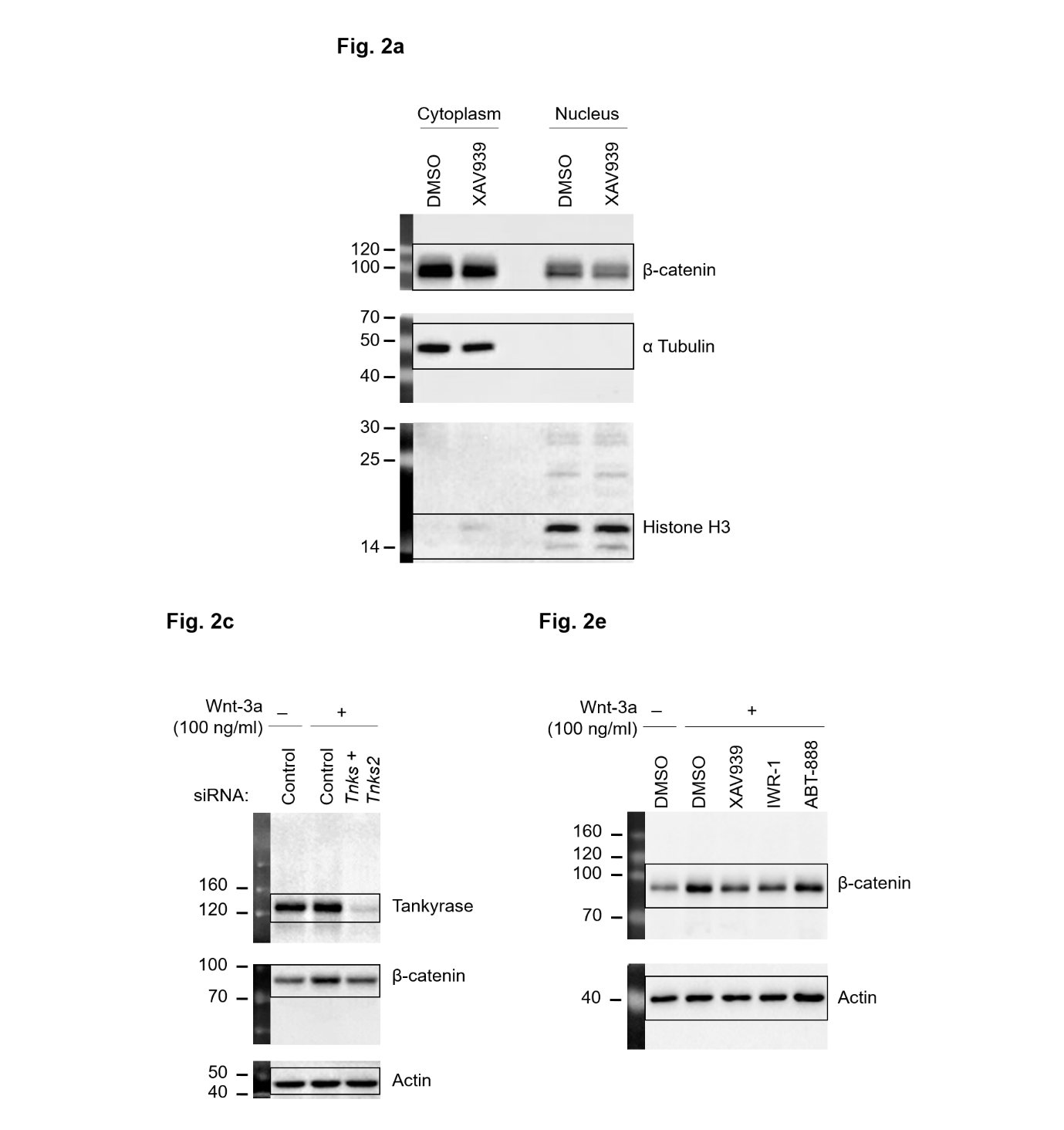
**

**Supplementary Fig. 9** Full-size immunoblot images (continued).

**
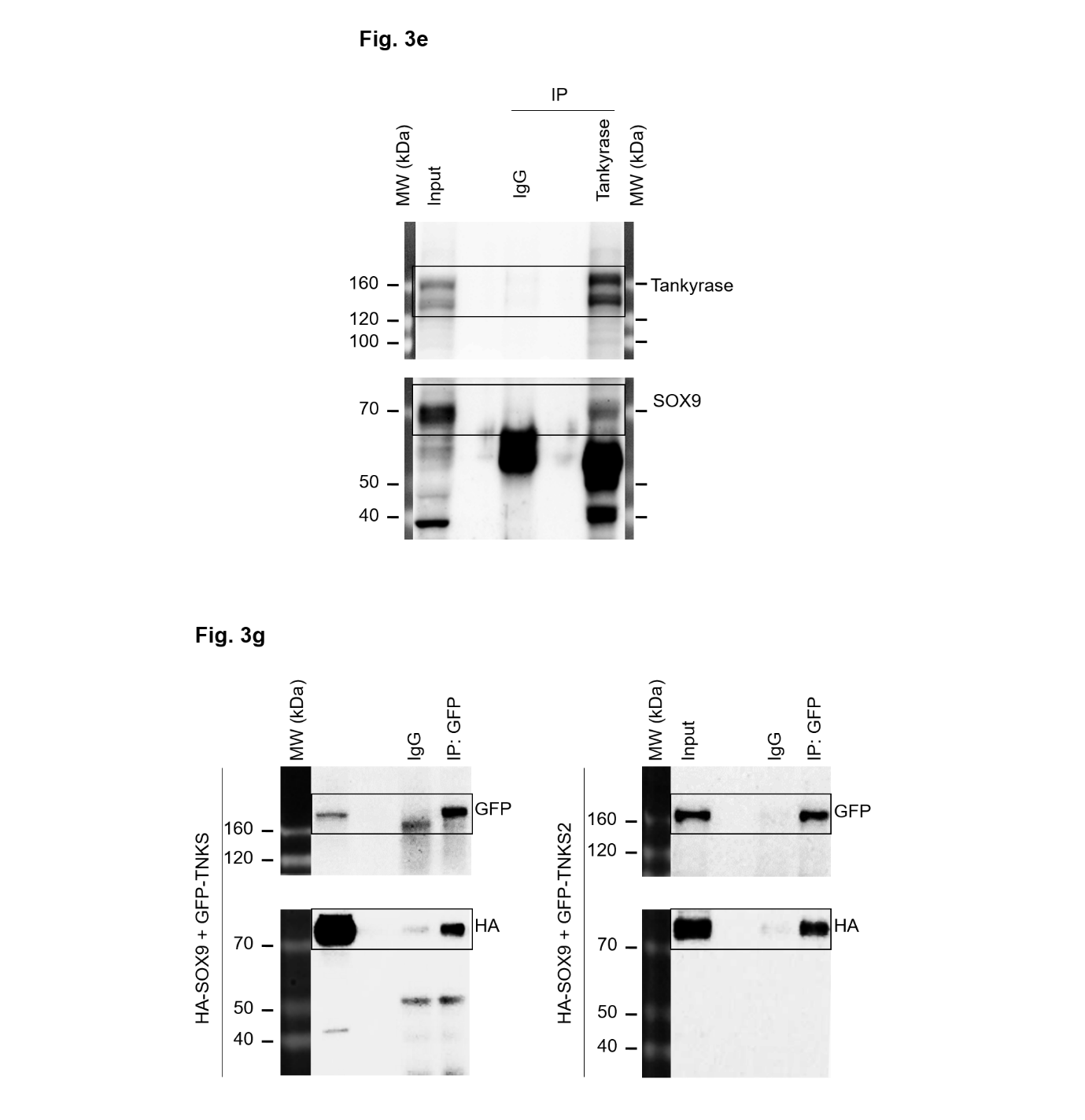
**

**Supplementary Fig. 9** Full-size immunoblot images (continued).

**
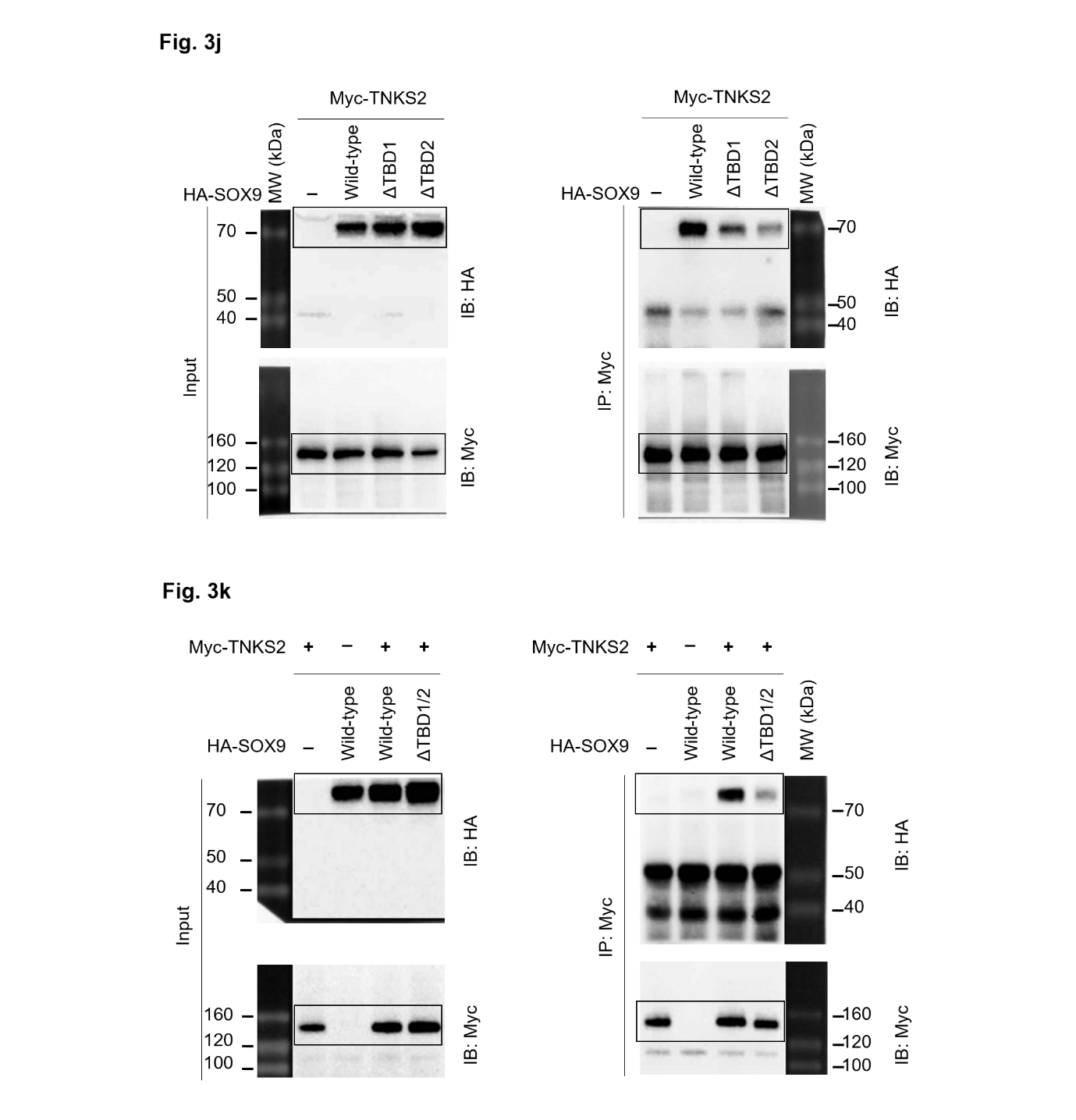
**

**Supplementary Fig. 9** Full-size immunoblot images (continued).

**
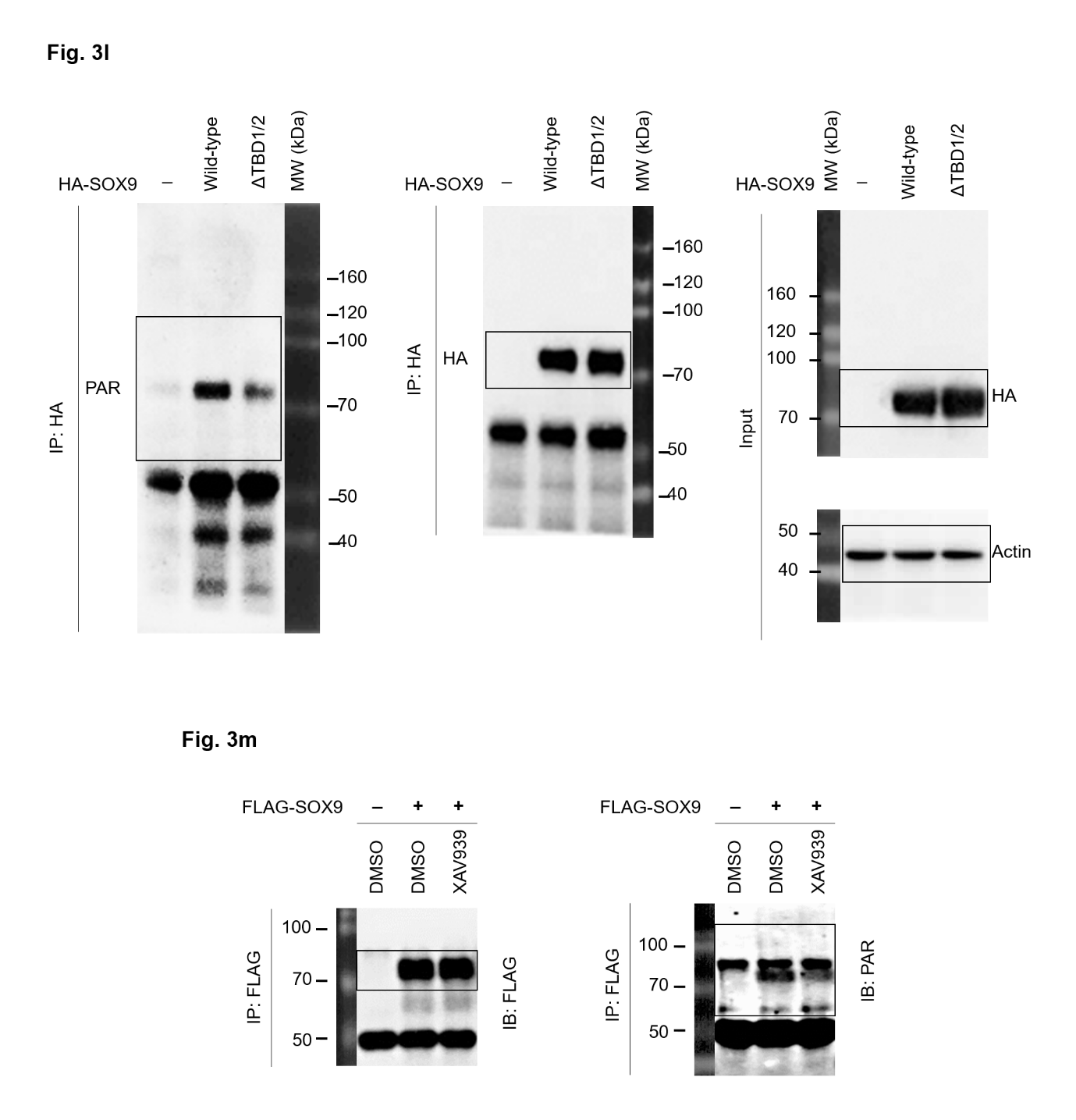
**

**Supplementary Fig. 9** Full-size immunoblot images (continued).


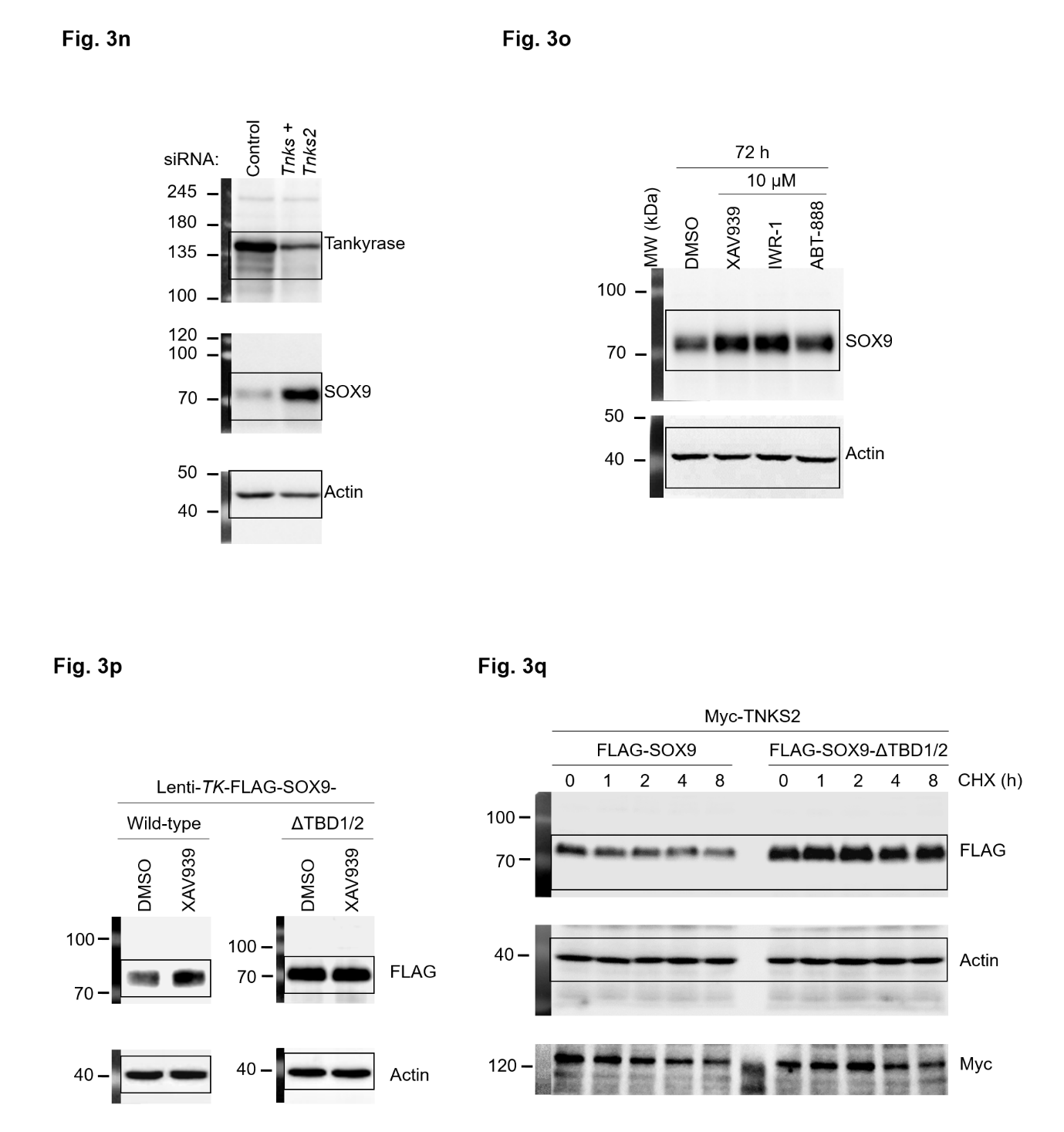


**Supplementary Fig. 9** Full-size immunoblot images (continued).


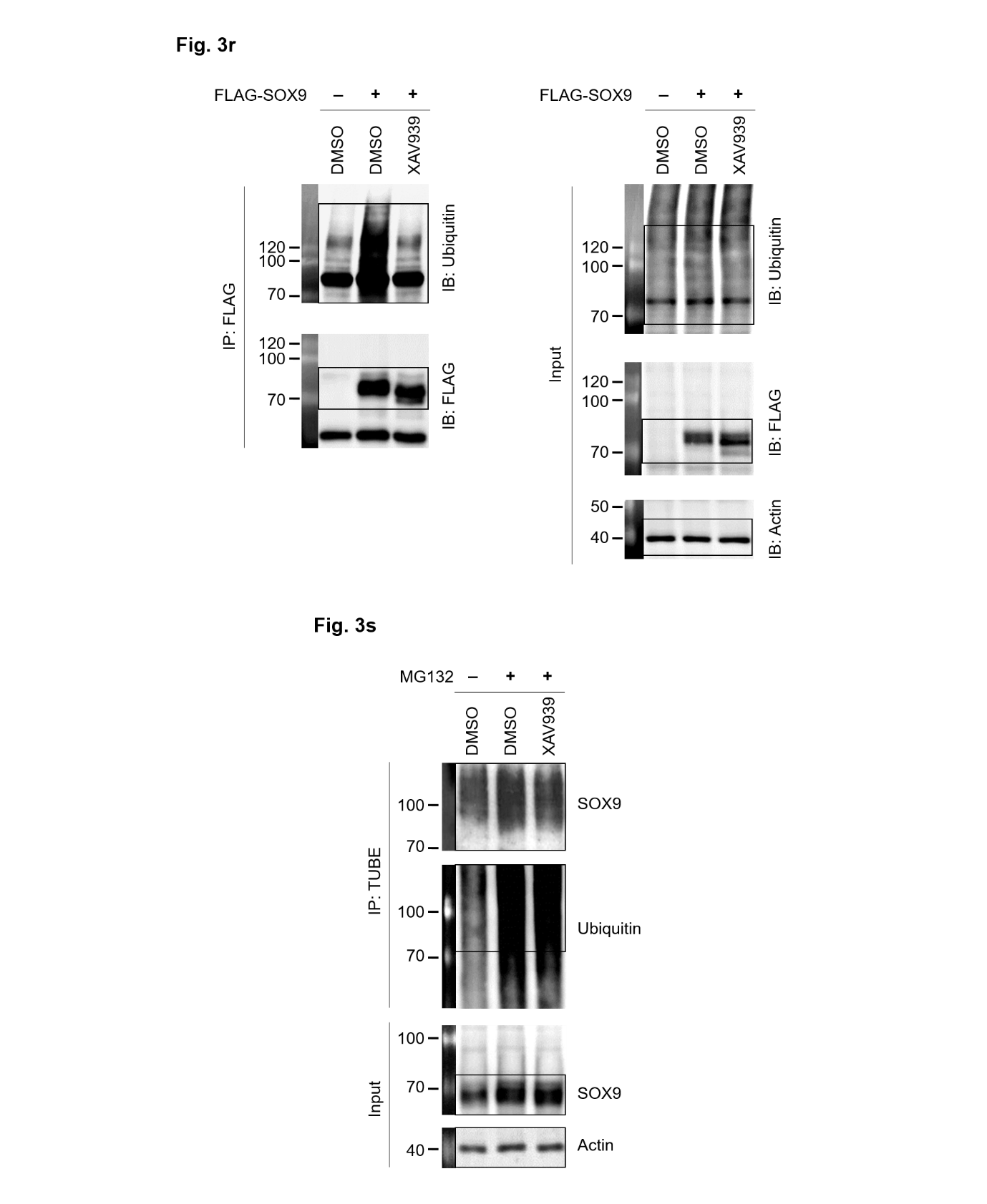


**Supplementary Fig. 9** Full-size immunoblot images (continued).

**
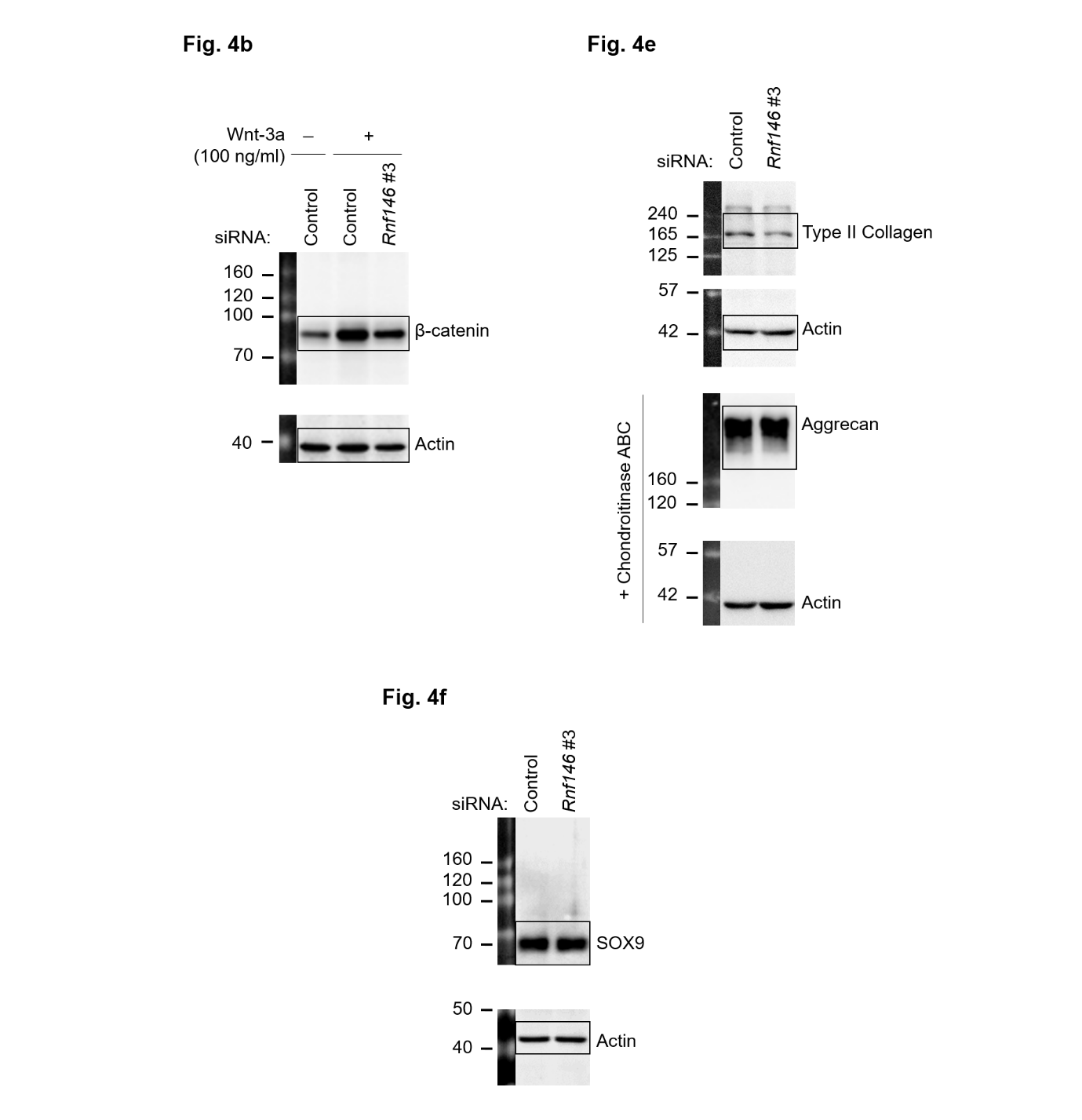
**

**Supplementary Fig. 9** Full-size immunoblot images (continued).


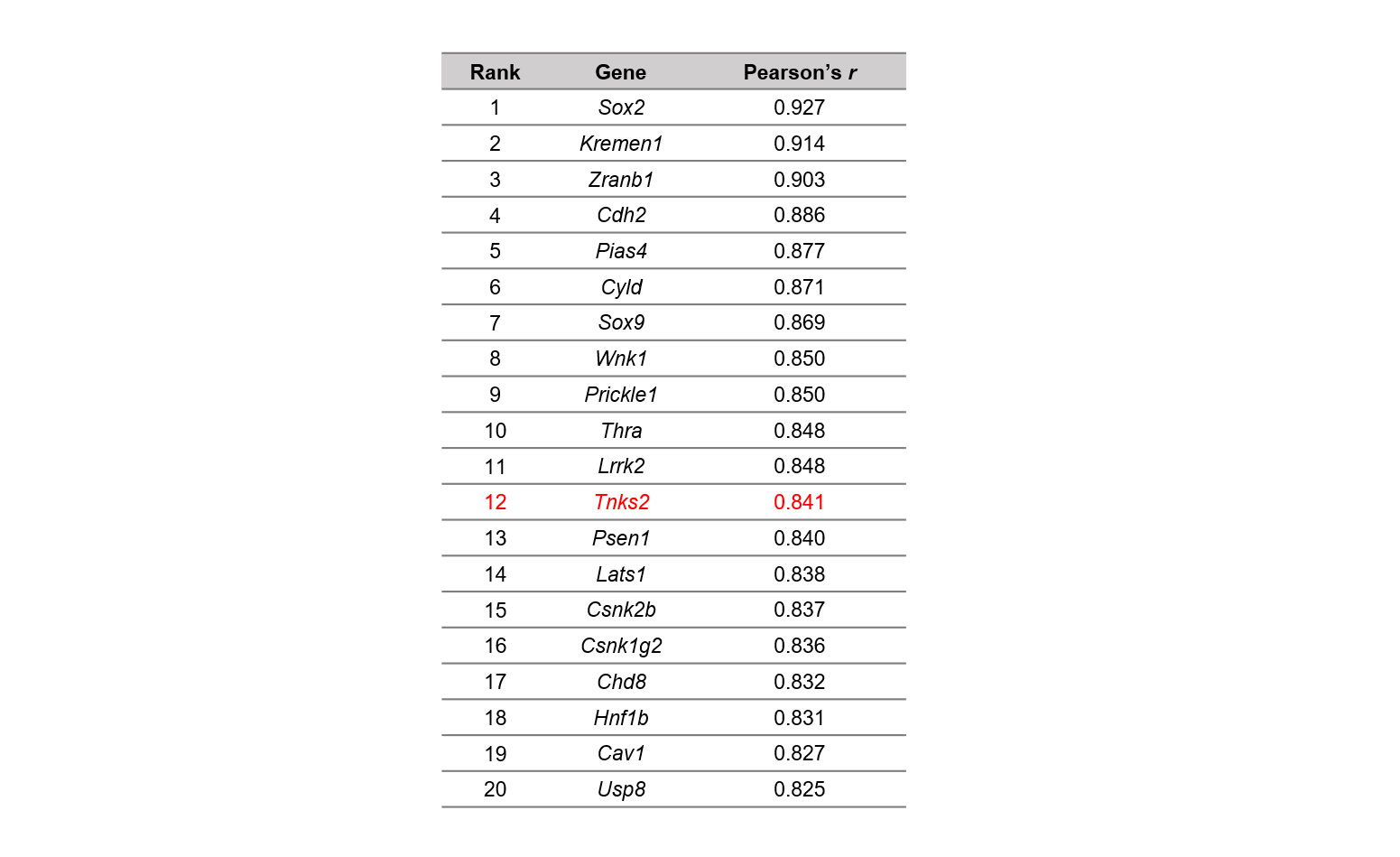
**Supplementary Table 1** Pearson’s correlation coefficients between Factor 1 and Wnt-related genes

*Mus musculus* genes in the Gene Ontology class “Wnt signaling pathway” were regarded as Wnt-related genes. Among the 431 genes in this category, 297 genes that were reliably detected in the cartilage tissues of 16 strains of BXD mice were considered for the correlation analysis.

| Gene | Strand | siRNA sequences | Species |
| --- | --- | --- | --- |
| *Tnks* #1 | S  AS | 5’-CACAGAGUCACACUGACUAdTdT-3’  5’-UAGUCAGUGUGACUCUGUGdTdT-3’ | Mouse |
| *Tnks* #2 | S  AS | 5’-GUCUGUCGUUGAGUACCUUdTdT-3’  5’-AAGGUACACAACGACAGACdTdT-3’ | Mouse |
| *Tnks* #3 | S  AS | 5’-ACAUAGCAGCGUUACUGAUdTdT-3’  5’-AUCAGUAACGCUGCUAUGUdTdT-3’ | Mouse |
| *Tnks2* #1 | S  AS | 5’-CAGUGUAGUUUUGAGUCUAdTdT-3’  5’-UAGACUCAAAACUACACUGdTdT-3’ | Mouse |
| *Tnks2* #2 | S  AS | 5’-CUGUUCUGACUGGUGACUAdTdT-3’  5’-UAGUCACCAGUCAGAACAGdTdT-3’ | Mouse |
| *Tnks2* #3 | S  AS | 5’-GUGUCUACUUGUAUCACAUdTdT-3’  5’-AUGUGAUACAAGUAGACACdTdT-3’ | Mouse |
| *Ctnnb1* #1 | S  AS | 5’-GUUUUAGGCCUGUUUGUAAdTdT-3’  5’-UUACAAACAGGCCUAAAACdTdT-3’ | Mouse |
| *Ctnnb1* #2 | S  AS | 5’-UCUGAACGUGCAUUGUGAUdTdT-3’  5’-AUCACAAUGCACGUUCAGAdTdT-3’ | Mouse |
| *Ctnnb1* #3 | S  AS | 5’-GUAAUCUGGAGACGUGUAAdTdT-3’  5’-UUACACGUCUCCAGAUUACdTdT-3’ | Mouse |
| *Rnf146* #1 | S  AS | 5’-CAGAUACCUCCGUUGAAGAdTdT-3’  5’-UCUUCAACGGAGGUAUCUGdTdT-3’ | Mouse |
| *Rnf146* #2 | S  AS | 5’-CUCUAGAGCAUCACAGCUUdTdT-3’  5’-AAGCUGUGAUGCUCUAGAGdTdT-3’ | Mouse |
| *Rnf146* #3 | S  AS | 5’-GUCGACAAGAGAUUCCUGAdTdT-3’  5’-UCAGGAAUCUCUUGUCGACdTdT-3’ | Mouse |
| *TNKS* | S  AS | 5’-GCAUGGAGCUUGUGUUAAUUU-3’  5’-AUUAACACAAGCUCCAUGCUU-3’ | Human |
| *TNKS2* | S  AS | 5’-GGAAAGACGUAGUUGAAUAUU-3’  5’-UAUUCAACUACGUCUUUCCUU-3’ | Human |
| *Sox9* #1 | S  AS | 5’-GUAAAGGAAGGUAACGAUUdTdT-3’  5’-AAUCGUUACCUUCCUUUACdTdT-3’ | Mouse |
| *Sox9* #2 | S  AS | 5’-GAGACAUCGGACAGACCUUdTdT-3’  5’-AAGGUCUGUCCGAUGUCUCdTdT-3’ | Mouse |
| *Sox9* #3 | S  AS | 5’-GUUUGUUUCCCUCUCCAAAdTdT-3’  5’-UUUGGAGAGGGAAACAAACdTdT-3’ | Mouse |

**Supplementary Table 2** List of siRNAs. Abbreviations: S, sense strand; AS, antisense strand.

| Gene | Strand | Primer sequences | Species |
| --- | --- | --- | --- |
| *Hprt* | S  AS | 5’-AGTCCCAGCGTCGTGATTAG-3’  5’-GTATCCAACACTTCGAGAGGTC-3’ | Mouse |
| *Tnks1* | S  AS | 5’-GAAGGAAGGAGAAGTTGCGG-3’  5’-AATGAAAGGAGAACCGTGGAAC-3’ | Mouse |
| *Tnks2* | S  AS | 5’-CGGCGTCTTCAACAGATACA-3’  5’-AGCCATCAACCATACCTTCAG-3’ | Mouse |
| *Col2a1* | S  AS | 5’-ACCTTGGACGCCATGAAAGT-3’  5’-CGGGAGGTCTTCTGTGATCG-3’ | Mouse |
| *Comp* | S  AS | 5’-GTAAACACCGCCACTGATGA-3’  5’-TGGGAGAAGCAGAAGACACC-3’ | Mouse |
| *Col9a2* | S  AS | 5’-GATGGGTCCTCGTGGCTAT-3’  5’-GTTCCCTTTGGGCCTGTTAT-3’ | Mouse |
| *Col6a3* | S  AS | 5’-TTATGGTGCTGATGTTGACTGG-3’  5’-ATTGCTGTTGGTTTGGTCGTT-3’ | Mouse |
| *Acan* | S  AS | 5’-CCCAAGCACAGAGGTAAACAG-3’  5’-CTCACATTGCTCCTGGTCTG-3’ | Mouse |
| *Dcn* | S  AS | 5’-AGGCTTCCTACTCGGCTGTGA-3’  5’-GTTCGGCGGCATTTGACTTT-3’ | Mouse |
| *Col6a1* | S  AS | 5’-TGAAAATGTGCTCCTGCTGTG-3’  5’-TGTCCCGTTGAGTGTCAGAA-3’ | Mouse |
| *Col9a1* | S  AS | 5’-AGCTGATGGATTAACAGGACC-3’  5’-TTCCCAGGGTCTCCAATAGG-3’ | Mouse |
| *Bgn* | S  AS | 5’-GCATTGAGATGGGCGGGAA-3’  5’-AGTAGGGCACAGGGTTGTTG-3’ | Mouse |
| *Chad* | S  AS | 5’-ACAACCGCCTGAACCAACT-3’  5’-GGGGAGGGATTCTGTGTCTT-3’ | Mouse |
| *Matn3* | S  AS | 5’-CAGTGTGAGGGGTTTCTG-3’  5’-AGCACCATAAGTTCATAGCC-3’ | Mouse |
| *Ctnnb1* | S  AS | 5’-CCACAGGATTACAAGAAGCGG-3’  5’-CCATTCCCACCCTACCAAGT-3’ | Mouse |
| *Rnf146* | S  AS | 5’-AGCACAGAGAATGAACCAGCA-3’  5’-TGAAGCACCCTTTACACACAGA-3’ | Mouse |
| *Sox9* | S  AS | 5’-AAGATGACCGACGAGCAGGA-3’  5’-ATGTGAGTCTGTTCCGTGGC-3’ | Mouse |
| *HPRT1* | S  AS | 5’-CCTGGCGTCGTGATTAGTG-3’  5’-CTTGCGACCTTGACCATCTTT-3’ | Human |
| *TNKS1* | S  AS | 5’-TCAGGGAACGATTTTGCTGGA-3’  5’-ACTCTGGGTATGCCTGTTCTC-3’ | Human |
| *TNKS2* | S  AS | 5’-GCGATACCCAAGGCAGACATT-3’  5’-AACAAGAGGGCAGAGCAGATGG-3’ | Human |

**Supplementary Table 3** List of PCR primers. Abbreviations: S, sense strand; AS, antisense strand.

| Gene | Strand | Primer sequences | Enzyme Sites | Species | Plasmid |
| --- | --- | --- | --- | --- | --- |
| *SOX9* | S  AS | 5’-CCGAATTCATGAATCTCCTGGACCCCTTC-3’  5’-CGTCTAGATCAAGGTCGAGTGAGCTGTGT-3’ | EcoRI  XbaI | Human | pcDNA3-HA-SOX9 |
| *SOX9* | S  AS | 5’-AAGAATTCGAATCTCCTGGACCCCTTCAT-3’  5’-CGTCTAGATCAAGGTCGAGTGAGCTGTGT-3’ | EcoRI  XbaI | Human | pCMV10-3xFLAG-SOX9 |
| *SOX9* | S  AS | 5’-AAGCTAGCAACCATGGACTACAAAGACCA-3’  5’-CGTCTAGATCAAGGTCGAGTGAGCTGTGT-3’ | NheI  XbaI | Human | pTK-3xFLAG-SOX9 |
| *SOX9* | S  AS | 5’-AACATATGAATGAGTCTTCGGACCTCGCG-3’  5’-CGTCTAGATCAAGGTCGAGTGAGCTGTGT-3’ | NdeI  XbaI | Human | pLVX-Puro-TK-3xFLAG-SOX9 |
| *TNKS2* | S  AS | 5’-AAAAGCTTGGATCATGTCGGGTCGCCGCTG-3’  5’-AAGGATCCTTATCCATCGACCATACCTTCAGGCCTCATAA-3’ | HindIII  BamHI | Human | pEGFP-TNKS2 |

**Supplementary Table 4** List of primers used for subcloning. Abbreviations: S, sense strand; AS, antisense strand.

| Gene | Strand | Primer sequences | Mutagenesis Site | Species |
| --- | --- | --- | --- | --- |
| *SOX9* | S  AS | 5’-CAGCCCCCTATCGACTTCCGCGA-3’  5’-CCCCTCTCGCTTCAGGTCAGCCT-3’ | ∆TBD1  772-792bp | Human |
| *SOX9* | S  AS | 5’-AGCAGCGACGTCATCTCCAACAT-3’  5’-GAAGTCGATAGGGGGCTGTCT-3’ | ∆TBD2  814-834bp | Human |
| *SOX9* | S  AS | 5’-AGCAGCGACGTCATCTCCAACAT-3’  5’-CCCCTCTCGCTTCAGGTCAGCCT-3’ | ∆TBD1/2  772-834bp | Human |
| *SOX9* | S  AS | 5’-CCCTTGCCAGAGGGGGGCA-3’  5’-TGCCCCCTCTCGCTTCAGGTCA-3’ | R257A | Human |
| *SOX9* | S  AS | 5’-GACGTGGACATCGGCGAGCTGA-3’  5’-TGCGAAGTCGATAGGGGGCTGTCT-3’ | R271A | Human |

**Supplementary Table 5** List of primers used for mutagenesis. Abbreviations: S, sense strand; AS, antisense strand.

| Gene | Strand | Primer sequences | Species |
| --- | --- | --- | --- |
| Control | S  AS | 5’- CCGGAAACAAGATGAAGAGCACCAACTCGAGTTGGTGCTCTTCATCTTGTTTTTTTTG -3’  5’- AATTCAAAAAAAACAAGATGAAGAGCACCAACTCGAGTTGGTGCTCTTCATCTTGTTT -3’ |  |
| *Tnks* | S  AS | 5’- CCGGGCTAGATGTGTTGGCTGATATCTCGAGATATCAGCCAACACATCTAGCTTTTTG -3’  5’- AATTCAAAAAGCTAGATGTGTTGGCTGATATCTCGAGATATCAGCCAACACATCTAGC -3’ | Mouse |
| *Tnks2* | S  AS | 5’- CCGGCATCGACACAAGCTGATTAAACTCGAGTTTAATCAGCTTGTGTCGATGTTTTTG -3’  5’- AATTCAAAAACATCGACACAAGCTGATTAAACTCGAGTTTAATCAGCTTGTGTCGATG -3’ | Mouse |
| *Rnf146* | S  AS | 5’- CCGGATTTCTGCCCACGTAACATTACTCGAGTAATGTTACGTGGGCAGAAATTTTTTG -3’  5’- AATTCAAAAAATTTCTGCCCACGTAACATTACTCGAGTAATGTTACGTGGGCAGAAAT -3’ | Mouse |
| *TNKS* | S  AS | 5’- CCGGGCCCATAATGATGTCATGGAACTCGAGTTCCATGACATCATTATGGGCTTTTTG -3’  5’- AATTCAAAAAGCCCATAATGATGTCATGGAACTCGAGTTCCATGACATCATTATGGGC -3’ | Human |
| *TNKS2* | S  AS | 5’- CCGGAAGGAAAGACGTAGTTGAATACTCGAGTATTCAACTACGTCTTTCCTTTTTTTG -3’  5’- AATTCAAAAAAAGGAAAGACGTAGTTGAATACTCGAGTATTCAACTACGTCTTTCCTT -3’ | Human |

**Supplementary Table 6** List of primers used for shRNA plasmid construction. Abbreviations: S, sense strand; AS, antisense strand.

| Proteins involved in chondrogenesis (52 proteins) | | | | | |
| --- | --- | --- | --- | --- | --- |
| ALG2 | CR3L2 | GRN | NFKB2 | Q9DAB5 | SOX12 |
| BMAL1 | CREB1 | GSK3A | NKX32 | REL | SOX4 |
| BMP2 | CTNB1 | GSK3B | PDGFA | RELB | SOX9 |
| BMP4 | CYR61 | HHAT | PER1 | RHOA | TF65 |
| BMR1B | DHH | HIF1A | PP2BA | SHH | TNF12 |
| CANB1 | ENPP1 | HMGB2 | PP2BB | SIR1 | VNN1 |
| CANB2 | FGF18 | IHH | PP2BC | SMAD3 | WNT3A |
| CBP | FGFR3 | NFAC3 | PRGC1 | SOMA |  |
| CHP1 | GDF5 | NFKB1 | PTHR | SOX11 |  |

**Supplementary Table 7** List of mouse proteins in the IPA *chondrogenesis* category.

| SOX9 target genes in chondrocytes (91 genes) | | | | | |
| --- | --- | --- | --- | --- | --- |
| *Acan* | *Col9a2* | *Fzd9* | *Mgp* | *Rab11fip4* | *Susd5* |
| *Aldh1l2* | *Col9a3* | *Gfpt1* | *Mia* | *Rhbdd1* | *Tprgl* |
| *Alx1* | *Colgalt2* | *Gls* | *Mtss1l* | *Rnf144a* | *Trib3* |
| *Arsi* | *Cox17* | *Got1* | *Ncmap* | *Rtkn* | *Trim47* |
| *Atf4* | *Cp* | *Grb2* | *Ndufa2* | *Scin* | *Trpv4* |
| *B230206H07Rik* | *Cpm* | *Hip1r* | *Oat* | *Sdk2* | *Ucma* |
| *B4galnt3* | *D630045J12Rik* | *Hr* | *Papss2* | *Slc1a5* | *Wscd2* |
| *Bcat1* | *Dnttip1* | *Kcns1* | *Pck2* | *Slc26a2* | *Wwp2* |
| *Bmp6* | *Enpp2* | *Lcn2* | *Pcolce2* | *Slc38a3* | *Xylt1* |
| *Chadl* | *Extl1* | *Ldlrad3* | *Pde4dip* | *Slc39a14* | *Zfp385b* |
| *Chst11* | *Fam89a* | *Lect1* | *Phyh* | *Smpd3* | *Zfp385c* |
| *Cmklr1* | *Fbxo7* | *Leprel1* | *Plxnb1* | *Snorc* |  |
| *Col11a1* | *Fgfr3* | *Lgals3* | *Ppp1r1b* | *Sobp* |  |
| *Col27a1* | *Fgfrl1* | *Loxl4* | *Ppp2ca* | *Sox6* |  |
| *Col2a1* | *Foxd1* | *Matn3* | *Prdx5* | *Spats2l* |  |
| *Col9a1* | *Fry* | *Mgat4a* | *Prelp* | *Stk39* |  |

**Supplementary Table 8** List of SOX9 targets in chondrocytes.

| Cartilage-signature genes (235 genes) | | | | | | | | |
| --- | --- | --- | --- | --- | --- | --- | --- | --- |
| *3632451O06Rik* | *Cd14* | *Dio2* | *Fzd9* | *Lect1* | *Nptx1* | *Scrg1* | *Sort1* | *Zdbf2* |
| *4930523C07Rik* | *Cdkn1a* | *Dnajb9* | *Gab1* | *Lipo3* | *Nr4a2* | *Scube3* | *Sox5* | *Zfp385b* |
| *A2m* | *Cgref1* | *Ecm2* | *Gfpt2* | *Loxl4* | *Nr4a3* | *Sdk2* | *Sox6* | *Zim1* |
| *Acan* | *Chac1* | *Edil3* | *Gjc3* | *Matn1* | *Nt5e* | *Sec16b* | *Sox9* |  |
| *Adamts3* | *Chad* | *Efcab1* | *Glis3* | *Matn3* | *Omd* | *Sema3e* | *Sparcl1* |  |
| *Adcy2* | *Chadl* | *Egr1* | *Gm22* | *Mdfi* | *Panx3* | *Sema6a* | *Srgap1* |  |
| *Adgrg1* | *Chrdl1* | *Egr2* | *Gm39701* | *Mertk* | *Papss2* | *Serinc5* | *Srgn* |  |
| *Airn* | *Chst11* | *Ehd3* | *Gm7265* | *Mfi2* | *Pcsk6* | *Sim2* | *Srxn1* |  |
| *Ak4* | *Clec3a* | *Eng* | *Gprc5a* | *Mfsd7c* | *Pde3a* | *Slc16a2* | *Stk26* |  |
| *Alx1* | *Cmklr1* | *Enpp1* | *Gpx3* | *Mgat4a* | *Perp* | *Slc16a4* | *Stk32b* |  |
| *Angptl1* | *Cmtm5* | *Enpp2* | *Grb10* | *Mia* | *Phxr4* | *Slc1a1* | *Stk40* |  |
| *Arc* | *Col10a1* | *Epas1* | *Gstk1* | *Mir377* | *Pla2g5* | *Slc1a5* | *Sulf2* |  |
| *Arl5b* | *Col11a1* | *Epyc* | *Hapln1* | *Mir411* | *Plcd1* | *Slc22a23* | *Tcn2* |  |
| *Asb4* | *Col11a2* | *Ern1* | *Hist1h1c* | *Mir505* | *Plet1* | *Slc22a4* | *Tet1* |  |
| *Atf3* | *Col2a1* | *Extl1* | *Hivep2* | *Mir568* | *Plod2* | *Slc25a36* | *Tet2* |  |
| *Atp1b2* | *Col9a1* | *F13a1* | *Hpgd* | *Moxd1* | *Prg4* | *Slc26a2* | *Tgfb2* |  |
| *Auts2* | *Col9a2* | *Fabp7* | *Igsf9b* | *Mpzl2* | *Prkg2* | *Slc2a10* | *Tmbim1* |  |
| *B4galnt3* | *Col9a3* | *Fam180a* | *Il16* | *Mt2* | *Prss35* | *Slc38a3* | *Tmem56* |  |
| *Baiap2l1* | *Colgalt2* | *Fam19a5* | *Islr* | *Mtap7d3* | *Ptger1* | *Slc6a12* | *Tnfrsf21* |  |
| *BC026585* | *Comp* | *Fam46a* | *Itga10* | *Mtss1l* | *Rab11fip4* | *Slc7a11* | *Tns2* |  |
| *Bdh1* | *Cpm* | *Fbln7* | *Kank1* | *Mustn1* | *Rbp4* | *Slc7a3* | *Tram2* |  |
| *Bmp2* | *Cpxm2* | *Fgfr2* | *Kcna6* | *Ncmap* | *Rcan1* | *Slc8a3* | *Trp53inp2* |  |
| *Bmp5* | *Creb3l2* | *Fgfr3* | *Kcnk1* | *Ndrg2* | *Rgs2* | *Smox* | *Trps1* |  |
| *Bmp6* | *Crispld1* | *Fmod* | *Kcnma1* | *Nebl* | *Rin2* | *Smpdl3a* | *Trpv4* |  |
| *Btg2* | *Cspg4* | *Fos* | *Kdm6b* | *Nfatc1* | *Rnf144b* | *Snora23* | *Ucma* |  |
| *C1qtnf3* | *Cthrc1* | *Fosb* | *Kdm7a* | *Nfatc2* | *S100a1* | *Snora28* | *Wisp3* |  |
| *C4b* | *Ctsh* | *Frmd4b* | *Kif21a* | *Ngf* | *S100b* | *Snorc* | *Xist* |  |
| *Car6* | *Cybrd1* | *Fry* | *Klhl13* | *Ninj1* | *Scara3* | *Snord82* | *Xylt1* |  |
| *Cd109* | *Cytl1* | *Frzb* | *Klk10* | *Ninj2* | *Scin* | *Sobp* | *Zbtb20* |  |

**Supplementary Table 9** List of cartilage-signature genes.

| Upregulated genes in osteoarthritic cartilage (150 genes) | | | | | |
| --- | --- | --- | --- | --- | --- |
| *3830406C13Rik* | *Cenpk* | *Fam167a* | *Kcnn4* | *Pcdh10* | *St6galnac5* |
| *Abracl* | *Cep55* | *Fam60a* | *Kcns3* | *Pcdh18* | *Stx1a* |
| *Adamts14* | *Chst13* | *Fat3* | *Kif20a* | *Pgm2l1* | *Syt11* |
| *Adamts5* | *Cited4* | *Fgf9* | *Lamb3* | *Plaur* | *Sytl2* |
| *Adamts6* | *Ckb* | *Fhl2* | *Lif* | *Plekhg1* | *Tbx3* |
| *Adgrg1* | *Clic3* | *Foxf1* | *Lmo2* | *Popdc3* | *Tenm3* |
| *Adtrp* | *Col13a1* | *Fstl3* | *Lrrc8c* | *Postn* | *Tfpi* |
| *AI661453* | *Col18a1* | *Fzd10* | *Lrrc8e* | *Prex2* | *Tgfbi* |
| *Akr1c20* | *Col1a1* | *Galnt7* | *Lum* | *Ptges* | *Tmem100* |
| *Anln* | *Col7a1* | *Gja1* | *Map1b* | *R3hdml* | *Tmem119* |
| *Arhgap44* | *Cpeb2* | *Gjb2* | *Mob3b* | *Rab23* | *Tmem200a* |
| *Arl4a* | *Csdc2* | *Glis3* | *Moxd1* | *Rcan1* | *Tmem59l* |
| *Arntl2* | *D330045A20Rik* | *Glrb* | *Msx2* | *Rhbdl2* | *Tnfaip6* |
| *Aspm* | *Diras1* | *Gmnn* | *Mtss1* | *S100a4* | *Tnfrsf12a* |
| *Aspn* | *Dkk3* | *Gpc4* | *Ncapg* | *Sema3c* | *Tom1l1* |
| *Atrnl1* | *Dnajc12* | *Gria2* | *Nedd4l* | *Serpine1* | *Top2a* |
| *B3gnt2* | *Dner* | *Hey2* | *Nedd9* | *Serpine2* | *Trim36* |
| *B3gnt5* | *Dsg2* | *Hhipl1* | *Ngf* | *Sgk1* | *Uroc1* |
| *Bmpr1b* | *Dusp4* | *Hmga2* | *Nt5e* | *Sik1* | *Vcan* |
| *C1galt1* | *Ebf3* | *Homer2* | *Ntf3* | *Slc2a5* | *Veph1* |
| *Car12* | *Egr2* | *Hunk* | *Ociad2* | *Slc38a5* | *Vwc2* |
| *Cdk1* | *Epha3* | *Ier3* | *Ogn* | *Slc6a6* | *Wisp1* |
| *Cdkn2b* | *Eva1a* | *Iqgap3* | *Osbpl3* | *Slitrk6* | *Wnt5a* |
| *Cdkn3* | *Evi2a* | *Itga3* | *P3h2* | *Sntb1* | *Zfp365* |
| *Cenpf* | *Fam132b* | *Kcne4* | *Pamr1* | *Sqrdl* | *Zfp367* |

**Supplementary Table 10** List of genes upregulated in osteoarthritic cartilage.

| Downregulated genes in osteoarthritic cartilage (71 genes) | | | | | |
| --- | --- | --- | --- | --- | --- |
| *Agtr2* | *Cmya5* | *Fbln7* | *Lgi4* | *Ptger3* | *Srl* |
| *Alx4* | *Col11a2* | *Fgf14* | *Lrrtm2* | *Rarres2* | *Steap4* |
| *Apol9b* | *Col16a1* | *Frzb* | *Mpped2* | *Rcan2* | *Stk32b* |
| *Atp1b2* | *Crim1* | *Gpc5* | *Myh14* | *Rflna* | *Tac1* |
| *C530008M17Rik* | *Cyp39a1* | *Gprc5b* | *Myoz3* | *Rspo3* | *Tceal5* |
| *Cacna1c* | *Dact1* | *Grin2c* | *Nfam1* | *Sdc3* | *Tmem176a* |
| *Cacna2d2* | *Dcc* | *Gucy1a3* | *Nrxn2* | *Sez6l* | *Tmem176b* |
| *Capn6* | *Ddit4* | *Hmgcll1* | *Obscn* | *Sgsm1* | *Tnfrsf4* |
| *Cdhr1* | *Erich3* | *Igf2* | *Pde3b* | *Slc14a1* | *Wnk2* |
| *Ces1a* | *Esr1* | *Il17rb* | *Piezo2* | *Slc25a27* | *Zcchc5* |
| *Chrdl2* | *Evx1* | *Il18bp* | *Ppp1r1b* | *Slitrk4* | *Zfp385c* |
| *Cmtm5* | *Fam198a* | *Kif1a* | *Prx* | *Sncg* |  |

**Supplementary Table 11** List of genes downregulated in osteoarthritic cartilage.
