# Supplementary material for: Tankyrase inhibition preserves osteoarthritic cartilage by coordinating cartilage matrix anabolism via effects on SOX9 PARylation
Source: Nat Commun. 2019 Oct 25;10:4898. doi: 10.1038/s41467-019-12910-2 (PMC6814715; doi:10.1038/s41467-019-12910-2)
Supplement: Supplementary file 4 — Reporting Summary [file 41467_2019_12910_MOESM4_ESM.pdf]

nature research

Corresponding author(s): Jin-Hong Kim

Last updated by author(s): Sep 23, 2019

Reporting Summary

Nature Research wishes to improve the reproducibility of the work that we publish. This form provides structure for consistency and transparency in reporting. For further information on Nature Research policies, see Authors & Referees and the Editorial Policy Checklist .

Statistics

For all statistical analyses, confirm that the following items are present in the figure legend, table legend, main text, or Methods section.

n/a	Confirmed	
☐	☒	The exact sample size (*n*) for each experimental group/condition, given as a discrete number and unit of measurement
☐	☒	A statement on whether measurements were taken from distinct samples or whether the same sample was measured repeatedly
☐	☒	The statistical test(s) used AND whether they are one- or two-sided<br>*Only common tests should be described solely by name; describe more complex techniques in the Methods section.*
☒	☐	A description of all covariates tested
☐	☒	A description of any assumptions or corrections, such as tests of normality and adjustment for multiple comparisons
☐	☒	A full description of the statistical parameters including central tendency (e.g. means) or other basic estimates (e.g. regression coefficient) AND variation (e.g. standard deviation) or associated estimates of uncertainty (e.g. confidence intervals)
☐	☒	For null hypothesis testing, the test statistic (e.g. *F*, *t*, *r*) with confidence intervals, effect sizes, degrees of freedom and *P* value noted<br>*Give P values as exact values whenever suitable.*
☒	☐	For Bayesian analysis, information on the choice of priors and Markov chain Monte Carlo settings
☐	☒	For hierarchical and complex designs, identification of the appropriate level for tests and full reporting of outcomes
☐	☒	Estimates of effect sizes (e.g. Cohen's *d*, Pearson's *r*), indicating how they were calculated

Our web collection on statistics for biologists contains articles on many of the points above.

Software and code

Policy information about availability of computer code

Data collection	*Provide a description of all commercial, open source and custom code used to collect the data in this study, specifying the version used OR state that no software was used.*
Data analysis	illuminaMousev1.db, lluminaMousev1p1.db, mouse4302.db, limma, biomaRt, Trimmomatic, TopHat, HTSeq, fastcluster, and DESeq2 R packages were used in this study. Cluster (http://bonsai.hgc.jp/~mdehoon/software/cluster/software.htm), Gitools, IBM SPSS Statistics, eulerAPE, ProteoWizard MSConvert, IPA, GalaxyPepDock, BIOVIA Discovery Studio Visualizer, Java Treeview, Enrichr, and GSEA were used in this study. ImageJ and Image-Pro Premier were used for image data quantification in this study.

For manuscripts utilizing custom algorithms or software that are central to the research but not yet described in published literature, software must be made available to editors/reviewers. We strongly encourage code deposition in a community repository (e.g. GitHub). See the Nature Research guidelines for submitting code & software for further information.

Data

Policy information about availability of data

All manuscripts must include a data availability statement. This statement should provide the following information, where applicable:

- Accession codes, unique identifiers, or web links for publicly available datasets
- A list of figures that have associated raw data
- A description of any restrictions on data availability

RNA-seq data will be deposited at GEO database.

Field-specific reporting

Please select the one below that is the best fit for your research. If you are not sure, read the appropriate sections before making your selection.

☒ Life sciences ☐ Behavioural & social sciences ☐ Ecological, evolutionary & environmental sciences

For a reference copy of the document with all sections, see nature.com/documents/nr-reporting-summary-flat.pdf

Life sciences study design

All studies must disclose on these points even when the disclosure is negative.

Sample size	Sample sizes were determined based on our previous experiences with cell culture and animal studies.
Data exclusions	No data were excluded from the analyses.
Replication	All attempts at replication were successful.
Randomization	Samples were randomly allocated to groups.
Blinding	To assess cartilage destruction, Safranin O stained samples were graded based on the Osteoarthritis Research Society International (OARSI) by three blinded observers. Cartilage regeneration was scored according to the International Cartilage Repair Society (ICRS) scoring system by three blinded observers.

Reporting for specific materials, systems and methods

We require information from authors about some types of materials, experimental systems and methods used in many studies. Here, indicate whether each material, system or method listed is relevant to your study. If you are not sure if a list item applies to your research, read the appropriate section before selecting a response.

Materials & experimental systems

n/a	Involved in the study	
☐	☒	Antibodies
☐	☒	Eukaryotic cell lines
☒	☐	Palaeontology
☐	☒	Animals and other organisms
☐	☒	Human research participants
☒	☐	Clinical data

Methods

n/a	Involved in the study	
☒	☐	ChIP-seq
☒	☐	Flow cytometry
☒	☐	MRI-based neuroimaging

Antibodies

Antibodies used	Anti-FLAG tag antibody (Cat No. F3165, clone M2) was purchased from Sigma-Aldrich. Antibodies against GFP (Cat No. sc-9996, clone B-2), Sox-9 (Cat No. sc-20095, clone H-90), Sox-9 (Cat No. sc-166505, clone E-9), Tankyrase-1/2 (Cat No. sc-8337, clone H-350), Tankyrase-1/2 (Cat No. sc-365897, clone E-10), Ubiquitin (Cat No. sc-8017, clone P4D1), Actin (Cat No. sc-1615, clone C-11), Histone H3 (Cat No. sc-517576, clone 1G1), and α Tubulin (Cat No. sc-23948, clone B-5-1-2) were purchased from Santa Cruz Biotechnology. Antibodies against aggrecan (Cat No. AB1031), type II collagen (Cat No. MAB8887, clone 6B3), and human mitochondria (Cat No. MAB1273, clone 113-1) were purchased from Millipore, and antibodies against Myc tag (Cat No. 2276, clone 9B11) and Sox9 (Cat No. 82630, clone D8G8H) were purchased from Cell Signaling Technology. Antibodies against HA tag (Cat No. ab9110) and MMP13 (Cat No. ab51072, clone EP1263Y) were purchased from Abcam. Anti-β-Catenin antibody (Cat No. 610154, clone 14) was obtained from BD Biosciences. Anti-Poly(ADP-ribose) antibody (Cat No. AG-20T-0001, clone 10H) was purchased from AdipoGen and was used. Anti-Poly(ADP-ribose) antibody (Cat No. 4335-AMC) was purchased from Trevigen.
Validation	Antibodies were validated via Western Blotting with the use of relevant positive controls.

Eukaryotic cell lines

Policy information about cell lines

Cell line source(s)	HEK293 and HEK293T cells are derived from human embryonic kidney cells.
Authentication	None of the cell lines used (HEK293, HEK293T) have been authenticated.
Mycoplasma contamination	The cell lines were not tested for mycoplasma contamination.

Commonly misidentified lines (See ICLAC register)	No commonly misidentified cell lines were used.

Animals and other organisms

Policy information about studies involving animals; ARRIVE guidelines recommended for reporting animal research

Laboratory animals	For primary culture of mouse articular chondrocytes, cells were isolated from femoral condyles and tibial plateaus of 4–5-day-old ICR mice. For micromass culture of mesenchymal cells, limb-bud cells were isolated from E11.5 ICR mouse embryos. Eight or twelve-week-old male ICR mice or twelve-week-old male C57BL/6 mice were used for experimental OA. Twelve-week-old male Sprague Dawley rats were used for an osteochondral defect model.
Wild animals	*Provide details on animals observed in or captured in the field; report species, sex and age where possible. Describe how animals were caught and transported and what happened to captive animals after the study (if killed, explain why and describe method; if released, say where and when) OR state that the study did not involve wild animals.*
Field-collected samples	*For laboratory work with field-collected samples, describe all relevant parameters such as housing, maintenance, temperature, photoperiod and end-of-experiment protocol OR state that the study did not involve samples collected from the field.*
Ethics oversight	*Identify the organization(s) that approved or provided guidance on the study protocol, OR state that no ethical approval or guidance was required and explain why not.*

Note that full information on the approval of the study protocol must also be provided in the manuscript.

Human research participants

Policy information about studies involving human research participants

Population characteristics	Human cartilage specimens were sourced from 5 osteoarthritis patients undergoing total knee replacement. (IRB No. 30-2017-48, IRB No. E1803/003-009). All participants are female and between 64 and 78 years of age.
Recruitment	*Describe how participants were recruited. Outline any potential self-selection bias or other biases that may be present and how these are likely to impact results.*
Ethics oversight	*Identify the organization(s) that approved the study protocol.*

Note that full information on the approval of the study protocol must also be provided in the manuscript.
