# Supplementary material for: Tankyrase inhibition preserves osteoarthritic cartilage by coordinating cartilage matrix anabolism via effects on SOX9 PARylation
Source: Nat Commun. 2019 Oct 25;10:4898. doi: 10.1038/s41467-019-12910-2 (PMC6814715; doi:10.1038/s41467-019-12910-2)
Supplement: Supplementary file 5 — Description of Additional Supplementary Files [file 41467_2019_12910_MOESM5_ESM.pdf]

**Title: Supplementary Data 1**

**Description:** List of tankyrase-binding peptides and proteins identified in mouse articular chondrocytes.

**Title: Supplementary Data 2**

**Description:** TTS scoring list for the 804 identified tankyrase-binding proteins in chondrocytes.
